# Back to Life, Back to Reality: Participation and Function After Acute Hospitalization in Older Adults

**DOI:** 10.1093/geroni/igab046.2081

**Published:** 2021-12-17

**Authors:** Maya Arieli, Racheli Kizony, Efrat Gil, Maayan Agmon

**Affiliations:** 1 Department of Occupational Therapy, University of Haifa, Israel, Haifa, HaZafon, Israel; 2 Clalit health services, Haifa and West Galilee, Israel, Haifa, HaZafon, Israel; 3 The Cheryl Spencer Department of Nursing, University of Haifa, Israel, Haifa, HaZafon, Israel

## Abstract

This study aims to describe and compare functional trajectories (i.e., participation versus basic daily function) from pre-hospitalization period to one and three-months post-discharge, among older adults hospitalized for acute medical illness, of two age groups: ages 65-75, n=39, >75, n=38. A Prospective longitudinal study was conducted, starting during hospitalization in internal ward and followed by home visits (1 month) and telephone interviews (3 months). Participation was measured by the Activity Card Sort (ACS) that queries about instrumental (e.g. shopping), leisure (e.g. physical activity), and social activities. Basic daily function was measured by the Modified Barthel Index (mBI). Wilcoxon test was used to compare between the ACS and mBI total retained scores within age groups. A mixed model repeated measures ANCOVA was used to compare time by group effects in ACS total scores. The results showed that basic function in both age groups was preserved, and both groups experienced a significantly greater decrease in participation level compared with basic function at one month (z=-4.1, p=.001, z=-4.5, p=.001) and at three months (z=-4.1, p=.001, z=-4.1, p=.001) in the "younger" and "older" groups, respectively. Participation trajectories were similar among age groups, however, the "older" group experienced a significantly greater decrease in participation (F(1)=(4.3), p=.042, η2= .056). Findings indicate that the traditional measure of basic function does not capture the broad spectrum of older adults’ full life and overshadows the reduced participation in meaningful activities. Health care professionals should adopt a comprehensive approach toward functional assessment to encompass participation beyond basic daily function.

